# Characterization of the airway microbiome in preterm infants with bronchopulmonary dysplasia

**DOI:** 10.3389/fcimb.2025.1654502

**Published:** 2025-10-13

**Authors:** Zhidan Bao, Limei Niu, Yizhe Ma, Xianhui Deng, Luchun Wang, Mingyan Tao, Renqiang Yu

**Affiliations:** ^1^ Department of Neonatology, Jiangyin People’s Hospital of Nantong University, Jiangyin, China; ^2^ Department of Neonatology, Affiliated Women’s Hospital of Jiangnan University, Wuxi Maternity and Child Health Care Hospital, Wuxi, China

**Keywords:** bronchopulmonary dysplasia, airway, microbiome, preterm infant, neonates

## Abstract

**Background and aims:**

Bronchopulmonary dysplasia (BPD) represents a persistent respiratory condition that primarily affects preterm infants, distinguished by abnormal lung development and function. Previous studies have indicated a significant association between the pulmonary microbiome and various respiratory diseases. This study aimed to compare the airway microbiome composition and its temporal changes in preterm infants with and without BPD.

**Methods:**

We conducted a cohort study involving 14 infants diagnosed with BPD and 10 preterm infants without BPD, all born at a gestational age (GA) < 32 weeks. Tracheal aspirates were collected on day 1 during intubation, as well as on days 7 and 14 following the intubation procedure. Subsequently, bacterial DNA was extracted, and the 16S rRNA genes were amplified and sequenced to characterize the airway microbiome.

**Results:**

The demographic and clinical features, such as gestational age, birth weight, and sex ratio, were similar across the groups. However, BPD infants required prolonged duration for Continuous Positive Airway Pressure (25.0 d vs 8.5 d, *P* = 0.001), oxygen therapy (38.0 d vs 20.5 d, *P* = 0.001), antibiotic treatment (9.5 d vs 4.5 d, *P* = 0.004), and prolonged hospital admissions (44.0 d vs 25.5 d, *P* = 0.002). Microbiome analysis revealed that the BPD infants exhibited reduced bacterial diversity at birth and a consistent pattern of diminished bacterial diversity over time compared to the non-BPD group, as indicated by a lower Shannon index. The BPD group also showed a distinct microbial community composition, with significant differences in β-diversity observed at day 14 post-incubation. At the phylum level, both groups exhibited an increase in *Firmicutes* in the first two weeks, while the BPD group showed a progressive decline in the relative abundance of *Bacteroidetes*. At the genus level, the BPD infants exhibited an increased proportion of *Streptococcus* and *Acinetobacter*, and a decreased abundance of *Prevotella* over time.

**Conclusions:**

These findings indicate that the airway microbiome in infants with BPD is characterized by reduced diversity and distinct microbial profiles, which may contribute to the pathogenesis of the disease. Understanding these microbiome dynamics may help develop targeted therapeutic strategies aimed at modulating the microbiome to prevent or mitigate BPD in preterm infants.

## Introduction

Bronchopulmonary dysplasia (BPD) is a major neonatal respiratory condition that mainly affects preterm infants and can lead to long-term respiratory complications and increased healthcare costs (Van de Loo et al., 2024). The burden of BPD extends beyond the individual, placing substantial economic strain on healthcare systems due to prolonged hospitalization, increased medical interventions, and the need for ongoing respiratory support (Bhandari and Alexiou, 2023). Current management strategies for BPD primarily rely on supportive care, such as mechanical ventilation, oxygen therapy, and surfactant administration (Van de Loo et al., 2024). However, these approaches are limited in their inability to target the underlying pathophysiological mechanisms and the variability in treatment responses among affected infants. Therefore, it is essential to understand the mechanisms that contribute to the development of BPD. The immune processes that drive disease progression remain poorly understood (Fang et al., 2024). In this context, the significance of airway microbiota has been proposed (Natalini et al., 2023). Once thought to be a sterile environment, the lungs are now recognized as being routinely exposed to a diverse array of microorganisms (Natalini et al., 2023).

In recent times, significant progress and the adoption of culture-independent molecular sequencing techniques, especially high-throughput innovations like next-generation sequencing, have markedly improved the capacity to dynamically analyze microbial communities located in diverse areas of the human body (El Saie et al., 2022). The microbiota within the pulmonary system is essential for sustaining the functional equilibrium of the lungs (Liu et al., 2022). Studies have revealed a strong link between the pulmonary microbiota and the onset and progression of various respiratory disorders (Liu et al., 2022). Evidence from human studies suggests that microbial exposures begin early in life, typically within days to weeks after birth, and play a crucial role in the maturation of the immune system (Mishra et al., 2021). However, the specific mechanisms by which alterations in the airway microbiome contribute to ongoing immune activation and the resultant lung injury are still not fully understood. The mechanisms that facilitate the interaction between the microbiome and immune responses in the airways have not been fully elucidated (Kayongo et al., 2022). The precise pathways through which modifications in the microbiome influence the onset of BPD are not yet thoroughly elucidated. Furthermore, the impact of microbiota on the underdeveloped lung and the associated risk for BPD remains controversial (Dong et al., 2022).

Previous studies has highlighted the potential role of the microbiome in the development and progression of BPD. However, the specific microbial characteristics and their evolution in infants with BPD, compared with those without, remain insufficiently explored (Staude et al., 2023). This study aimed to fill this knowledge by investigating the microbiome features and their dynamics in infants diagnosed with BPD, contributing to a deeper understanding of the disease and potential avenues for targeted interventions. A comparative cohort design was employed to examine the microbiome characteristics and clinical features of infants diagnosed with BPD in contrast to a control group of infants without BPD. The primary objective of this pilot research was to characterize the longitudinal development of the airway microbiome (at days 1, 7, and 14 after intubation) in preterm infants with and without BPD. By integrating clinical data with microbiome analysis, this study aimed to identify specific microbial taxa that may serve as biomarkers for BPD, ultimately supporting improved diagnostic and therapeutic strategies for affected infants.

## Methods

### Subjects

This observational cohort study included preterm infants who were born at a gestational age (GA) ranging from 26 to 32 weeks; subsequently admitted to the neonatal intensive care unit (NICU) within 2 hours after birth; diagnosed with neonatal respiratory distress syndrome; who need tracheal intubation and mechanical ventilation at Jiangyin People’s Hospital of Nantong University. All participating infants were monitored until they reached 36 weeks of postmenstrual age (PMA). At that time, the physiological criteria for BPD according to the National Institute of Child Health and Human Development in 2018 (Higgins et al., 2018) were employed to categorize them into two groups: the BPD group (who developed BPD) and the non-BPD group (who did not develop the condition). Infants with major congenital anomalies (such as congenital heart disease, digestive tract malformations), a diagnosis of sepsis, or those who died before reaching 36 weeks’ PMA and whose hospital stay less than 14 days were excluded from the study. Clinical data were obtained from obstetric and neonatal records during the participants routine clinical care in the hospital. Informed consent was obtained from the parents of all infants. The sample size was determined by the number of eligible infants admitted to the NICU during the study period who met the clinical criteria for BPD diagnosis and had available samples. The study was approved by the Institutional Ethics Committee of Jiangyin People’s Hospital (Approval number: 2023-046).

### Tracheal aspirates sample collection

Tracheal aspirates (TA) samples were obtained at intubation within six hours post-delivery, prior to the administration of surfactant and at seven and fourteen days after intubation. And the TA samples were stored at -80°C until utilized.

### Bioinformatics analysis

The methodology for 16S rRNA sequencing utilized in the analysis of the microbiome present in the TA samples has been described in detail in prior studies (Lal et al., 2018). The sequencing library was constructed using the MetaVX Library Preparation Kit. Briefly, 20–50 ng of DNA was used to generate amplicons that target the V3 and V4 hypervariable regions of the bacterial 16S rRNA gene. The forward primersequence was “ACTCCTACGGGAGGCAGCAG”, and the reverse primer sequence was “GGACTACHVGGGTWTCTAAT”. Paired end reads were assembled and analyzed with VSEARCH (v2.14.1) after demultiplexing and primer trimming (Rognes et al., 2016). Low-quality reads were filtered and chimeras removed using UCHIME in *de-novo* and reference modes. Exact amplicon sequence variants (ASV) were resolved with the UNOISE-like denoising implemented in VSEARCH. Taxonomy was assigned using the SINTAX classifier (USEARCH v10; -strand both, -sintax_cut-off 0.6) against the rdp_16s_v18 reference database. ASV tables were analyzed without rarefaction; relative abundances were used for compositional summaries. Alpha-diversity was computed in VSEARCH, where Shannon’s index reflects both richness and evenness, and Simpson’s index emphasizes dominance/evenness. Principal coordinates analysis (PCoA) was employed to assess the composition of global microbiota (β-diversity), utilizing Bray-Curtis dissimilarities. Differentially represented taxa between groups were identified with LEfSe (default settings unless otherwise specified) (Segata et al., 2011). Functional profiling was performed with PICRUSt2 to infer MetaCyc pathway abundances using the default pipeline (Douglas et al., 2020). Multiple testing was controlled by the Benjamini Hochberg procedure, and FDR-adjusted P values are reported where applicable.

### Statistical analysis

All statistics were performed utilizing R version 4.2.1 using standard functions and packages. Data are presented as the means ± standard deviation or median (P25, P75). For categorical variables, the Chi-Square Test and Fisher’s Exact Test were employed as appropriate. A *P* -value of less than 0.05 was considered statistically significant.

## Results

### Characteristics of infants with BPD and without BPD

The study included a cohort of 14 infants diagnosed with BPD and a non-BPD group of 10 preterm infants without BPD. Demographic and clinical characteristics of both groups are detailed in **Tables 1**, **2**. The incidence of maternal diseases, including preeclampsia, gestational diabetes mellitus, intrahepatic cholestasis of pregnancy, chorioamnionitis, prenatal antibiotic use, prenatal steroid treatment, and caesarean section was comparable across both groups. GA, birth weight (BW), birth height, birth head circumference, sex distribution, 1 min Apgar scores and 5 min Apgar scores did not show significant differences between the two groups.

**Table 1:** The clinical characteristics of the BPD and non-BPD infants.

Variables	Non-BPD (*n* = 10)	BPD (*n* = 14)	*P*-value
Maternal features			
Preeclampsia, n (%)	3 (30%)	6 (42.90%)	0.680
GDM, n (%)	4 (40%)	5 (35.70%)	0.999
Chorioamnionitis, n (%)	1 (10.0%)	0 (0.0%)	0.420
ICP, n (%)	1(10%)	1 (7.10%)	0.999
Prenatal antibiotic use (n, %)	6 (60%)	6 (42.9%)	0.680
Prenatal steroid treatment (n, %)	6 (60%)	6 (42.9)	0.680
Caesarean section, n (%)	2 (20.0%)	5 (35.7%)	0.650
Infant features			
Gestational age, weeks	30.96 ± 2.40	31.17 ± 1.44	0.790
Birth weight, g	1185.0 (1072.5, 1335.0)	1055.0 (993.5, 1437.5)	0.710
Birth height	40.5(40.0,42.0)	39.5(36.8,41.0)	0.120
Birth head circumference	30.1(28.75,32.25)	29.2(27.63,29.63)	0.120
Male, n (%)	6 (60.0%)	10 (71.4%)	0.670
1min Apgar scores	7.80 ± 1.75	8.00 ± 1.96	0.790
5 min Apgar scores	8.80 ± 0.79	8.86 ± 0.77	0.860

BPD, bronchopulmonary dysplasia; GDM, Gestational diabetes; ICP, intrahepatic cholestasis of pregnancy.

**Table 2:** The treatment and prognosis of the BPD and non-BPD infants.

Variables	Non-BPD (n=10)	BPD (n=14)	*P*-value
antibiotic use (d)	4.50 (3.75, 7.25)	9.5 (6.75,13.25)	0.004
TPN (d)	11.50 (10.00,15.50)	16.00 (13.00,18.25)	0.045
Ventilator days (d)	0.5 (0.00,4.00)	0 (0,5.50)	0.890
CPAP (d)	8.50 (3.75,17.00)	25.00 (18.00,39.00)	0.001
HFNC (d)	10.00 (8.50,15.50)	11.00 (5.50,13.25)	0.806
Oxygen days (d)	20.50 (12.25,28.25)	38.00 (32.00,75.5)	0.001
Caffeine use, n (%)	6(60%)	7(50%)	0.628
pulmonary surfactant	7(70%)	8(57.1%)	0.521
RDS, n (%)	10 (100%)	14 (100%)	–
hsPDA, n (%)	2(20%)	3(21.4%)	0.932
Hospital stays (d)	25.50 (16.25, 30.50)	44.00 (35.25, 56.50)	0.002
Head circumference at discharge (cm)	33(31.5,34.5)	33 (32,33.5)	0.980
Weight at discharge (kg)	2.28 ± 0.18	2.59 ± 0.29	0.010
Height at discharge (cm)	45.75 (45.0,46.0)	46.50 (45.0,47.0)	0.140

TPN, Total Parenteral Nutrition; CPAP, Continuous Positive Airway Pressure; HFNC, High-flow nasal cannula; RDS, neonatal respiratory distress syndrome; hsPDA, Hemodynamically Significant Patent Ductus Arteriosus.

However, infants predisposed to BPD required more days of respiratory support, indicated by increased Continuous Positive Airway Pressure days (25.0 d vs 8.5 d, *P* = 0.001) and longer oxygen utilization (38.0 d vs 20.5 d, *P* = 0.001), consistent with established risk factors for BPD. Infants diagnosed with BPD exhibited a longer duration of antibiotic administration (9.5 d vs 4.5 d, *P* = 0.004), extended utilization of total parenteral nutrition (16.0 d vs 11.5 d, *P=*0.045), and prolonged hospital admissions (44.0 d vs 25.5 d, *P* = 0.002). Moreover, the body weights at discharge in the BPD group were higher than the non-BPD group (2.59 vs 2.28 kg, *P* = 0.010).

### Distinct microbiome profiles in infants with and without BPD

**Figure 1:**
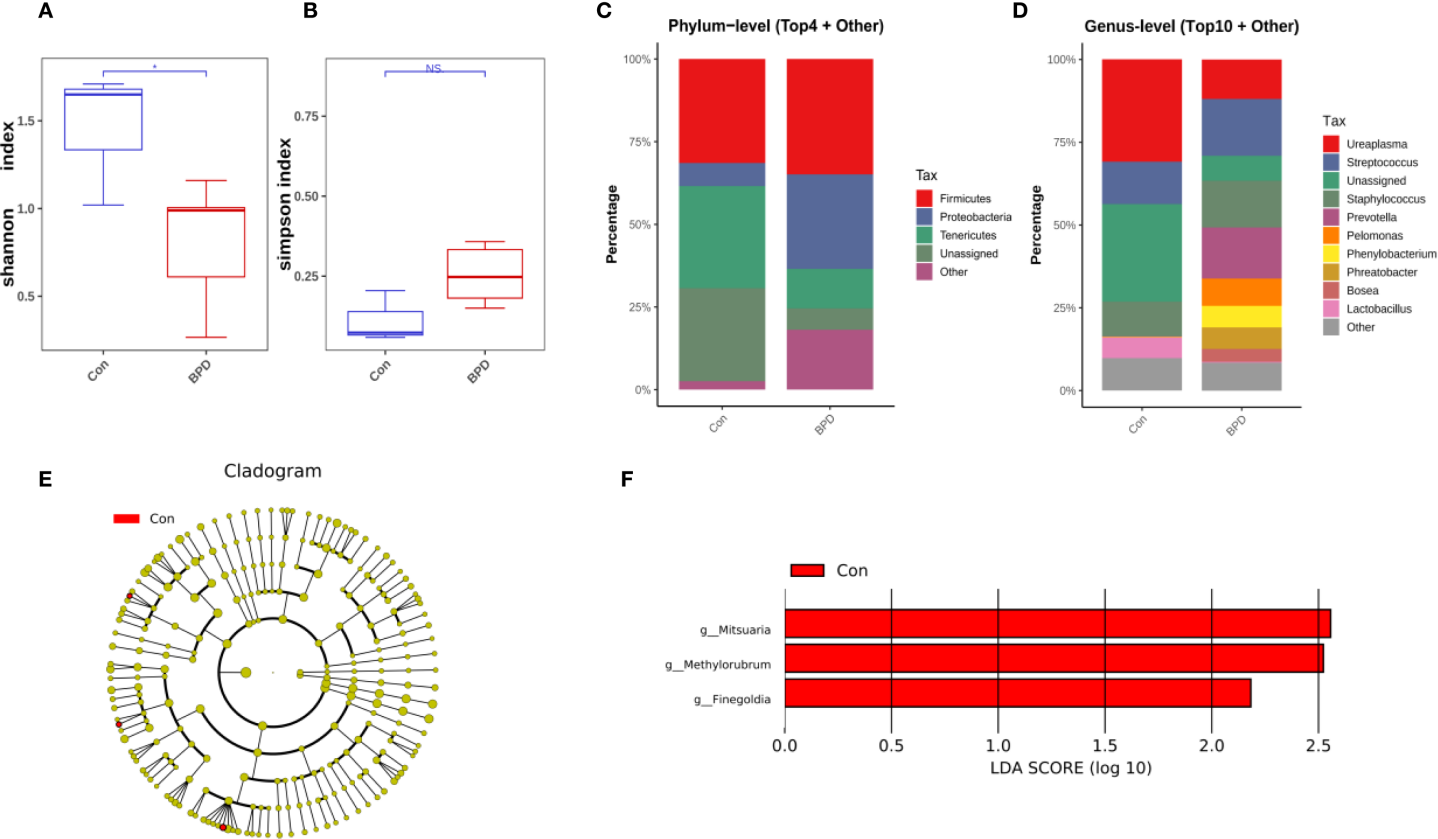
Microbiome features at intubation differ between infants with and without BPD. **(A)** Box plots of Shannon index of the BPD group and the non-BPD group at intubation; **(B)** simpson index of the BPD group and the non-BPD group at intubation; **(C)** The four species with the highest abundance at the phylum level were identified to create a histogram representing their relative abundance of the BPD group and the non-BPD group at intubation; **(D)** The top ten species with the highest abundance at the genus level were identified to create a histogram representing their relative abundance of the BPD group and the non-BPD group at intubation; **(E)** The cladogram constructed from representative sequences with branch and red colors indicating the species with significant difference; **(F)** Linear discriminant analysis effect size (LEfSe) analysis demonstrated LDA scores of the species with significant difference between the two groups at intubation. *P < 0.05. **P < 0.01.

At the time of intubation, neonates who later developed BPD exhibited reduced bacterial diversity, as evidenced by lower Shannon diversity index values compared with those without BPD (**Figure 1A**). In contrast, the community evenness, as indicated by the Simpson index, appeared comparable between the two groups (**Figure 1B**). Qualitative PCoA analysis was performed to evaluate β-diversity. No significant difference in microbial community composition was observed between BPD infants and the non-BPD group (*P* = 0.101).

According to the findings from species annotation, the species with the highest abundance at both the phylum and genus levels were identified to create a histogram representing their relative abundance. This visualization aimed to highlight the species with the highest relative abundance and their respective proportions across various taxonomic classifications. The analysis revealed that in infants who later progressed to BPD, *Firmicutes* and *Proteobacteria* were the most abundant at the phylum level, while *Firmicutes* and *Tenericutes* were the most abundant in preterm infants who did not develop BPD, as illustrated in **Figure 1C**. At the genus level, *Ureaplasma* was the most dominant genus in the non-BPD group, along with *Staphylococcus*, *Streptococcus*, and *Lactobacillus*. In contrast, the genera identified in the BPD cohort were more evenly distributed, with the early airway microbiome being dominated by *Staphylococcus*, *Ureaplasma*, *Streptococcus*, and *Prevotella* (**Figure 1D**). At the level of individual ASV, 16S rRNA sequences associated with *Mitsuaria* (*Proteobacteria*), *Finegoldia* (*Firmicutes*), and *Methylorubrum* (*Proteobacteria*) showed significantly decreased abundance in the BPD group compared with the non-BPD group (**Figure 1E**). Linear discriminant analysis effect size (LEfSe) analysis demonstrated LDA scores (**Figure 1F**), further supporting the identification of these taxa as relevant biomarkers for their respective groups.

**Figure 2:**
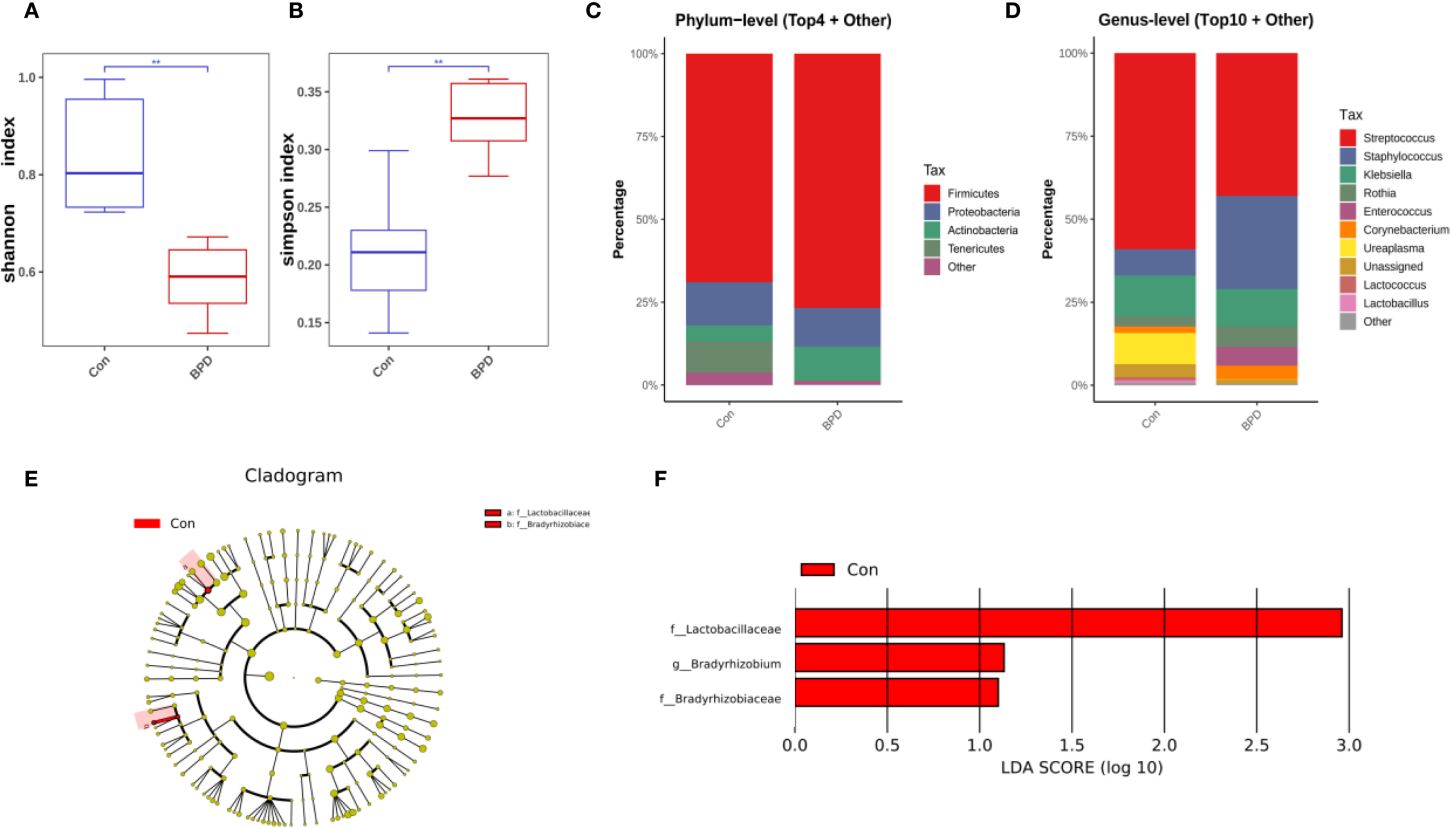
Microbiome features at day 7 post-intubation differ between infants with and without BPD. **(A)** Box plots of Shannon index of the BPD group and the non-BPD group at day 7 post-intubation; **(B)** simpson index of the BPD group and the non-BPD group at day 7 post-intubation; **(C)** The four species with the highest abundance at the phylum level were identified to create a histogram representing their relative abundance of the BPD group and the non-BPD group at day 7 post-intubation; **(D)** The top ten species with the highest abundance at the genus level were identified to create a histogram representing their relative abundance of the BPD group and the non-BPD group at day 7 post-intubation; **(E)** The cladogram constructed from representative sequences with branch and red colors indicating the species with significant difference; **(F)** Linear discriminant analysis effect size (LEfSe) analysis demonstrated LDA scores of the species with significant difference between the two groups at day 7 post-intubation. *P < 0.05. **P < 0.01.

On day 7 post-intubation, the BPD group still exhibited reduced bacterial diversity, as evidenced by a lower Shannon diversity index (**Figure 2A**). No significant difference in microbial community composition was observed between BPD infants and the non-BPD group based on PCoA analysis (*P* = 0.670). However, the BPD group was characterized by a significantly higher Simpson index compared with the non-BPD group, which may be associated with the increased number of some dominant strains in the non-BPD group (**Figure 2B**). On day 7, *Firmicutes* was the most abundant phylum in both groups of infants, as illustrated in **Figure 2C**. At the genus level, *Streptococcus* was the most dominant genus in both groups, while Staphylococcus was also a dominant genus in the BPD cohort (**Figure 2D**). The relative abundance of 16S rRNA genes from *Lactobacillaceae* (*Firmicutes*), *Bradyrhizobium* (*Proteobacteria*), and *Bradyrhizobiaceae* (*Proteobacteria*) was statistically lower in the BPD group at the ASV level (**Figures 2E, F**).

**Figure 3:**
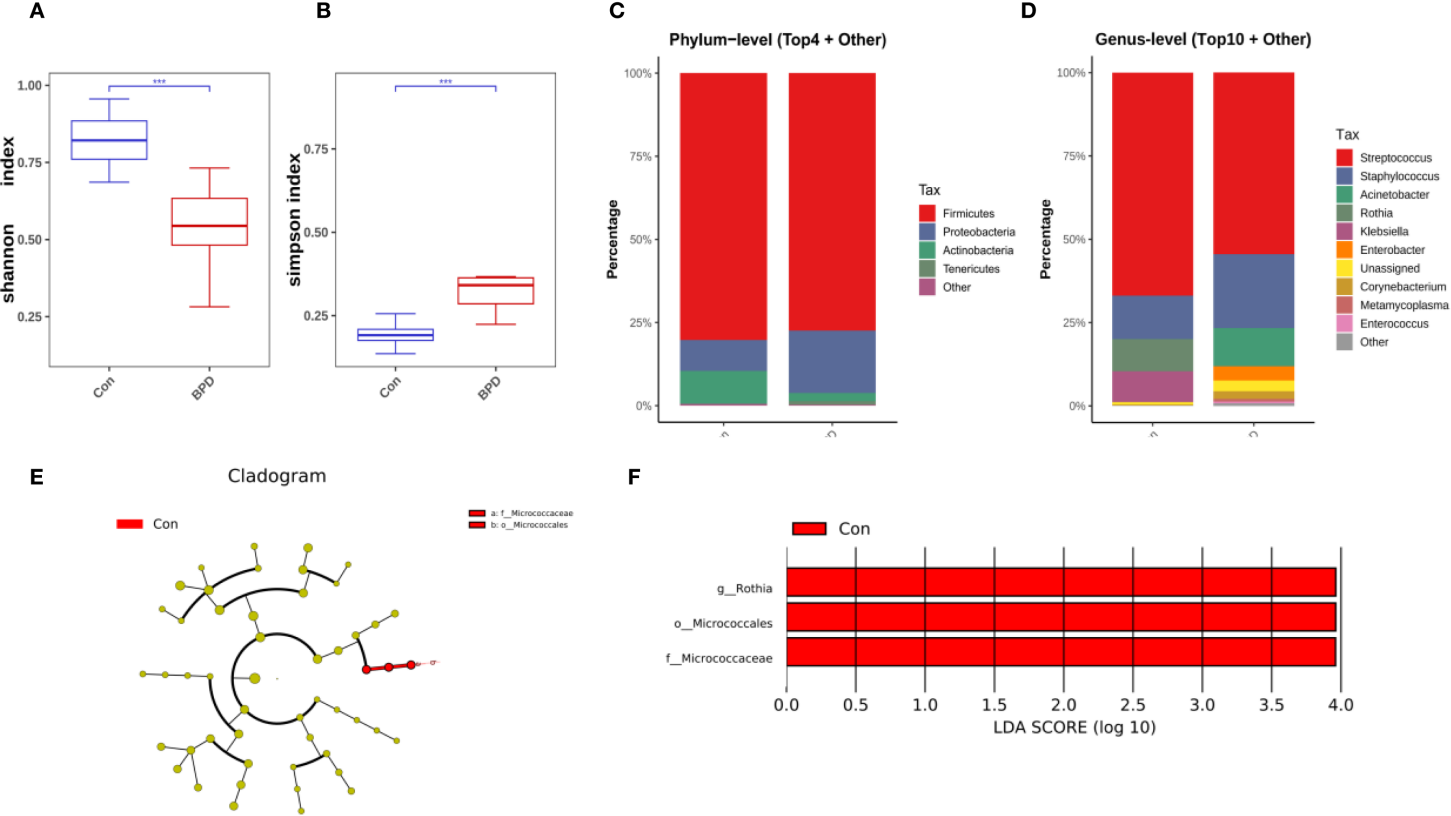
Microbiome features at day 14 post-intubation differ between infants with and without BPD. **(A)** Box plots of Shannon index of the BPD group and the non-BPD group at day 14 post-intubation; **(B)** simpson index of the BPD group and the non-BPD group at day 14 post-intubation; **(C)** The four species with the highest abundance at the phylum level were identified to create a histogram representing their relative abundance of the BPD group and the non-BPD group at day 14 post-intubation; **(D)** The top ten species with the highest abundance at the genus level were identified to create a histogram representing their relative abundance of the BPD group and the non-BPD group at day 14 post-intubation; **(E)** The cladogram constructed from representative sequences with branch and red colors indicating the species with significant difference; **(F)** Linear discriminant analysis effect size (LEfSe) analysis demonstrated LDA scores of the species with significant difference between the two groups at day 14 post-intubation. *P < 0.05. **P < 0.01.

On day 14 post-intubation, neonates who subsequently developed BPD demonstrated a decrease in bacterial diversity, as indicated by a lower Shannon diversity index but a significantly higher Simpson index compared with those without BPD (**Figures 3A, B**). The overall microbial community composition of BPD infants was also different from that of the non-BPD group, as indicated by PCoA analysis (*P* = 0.006). On day 14, *Firmicutes* remained the most abundant phylum in both groups, as illustrated in **Figure 3C**. At the genus level, *Stenotrophomonas*, *Staphylococcus*, and *Rothia* dominated the airway microbiota in the non-BPD group, whereas *Stenotrophomonas*, *Staphylococcus*, and *Acinetobacter* were the most abundant genera in the BPD group (**Figure 3D**). A marked increase in the relative abundance of *Rothia* (*Actinobacteria*), *Micrococcales* (*Actinobacteria*), and *Micrococcaceae* (*Actinobacteria*) was observed in the non-BPD group at the ASV level (**Figures 3E, F**), which partially explains the lower Simpson index.

### Microbiome dynamics in infants with and without BPD

**Figure 4:**
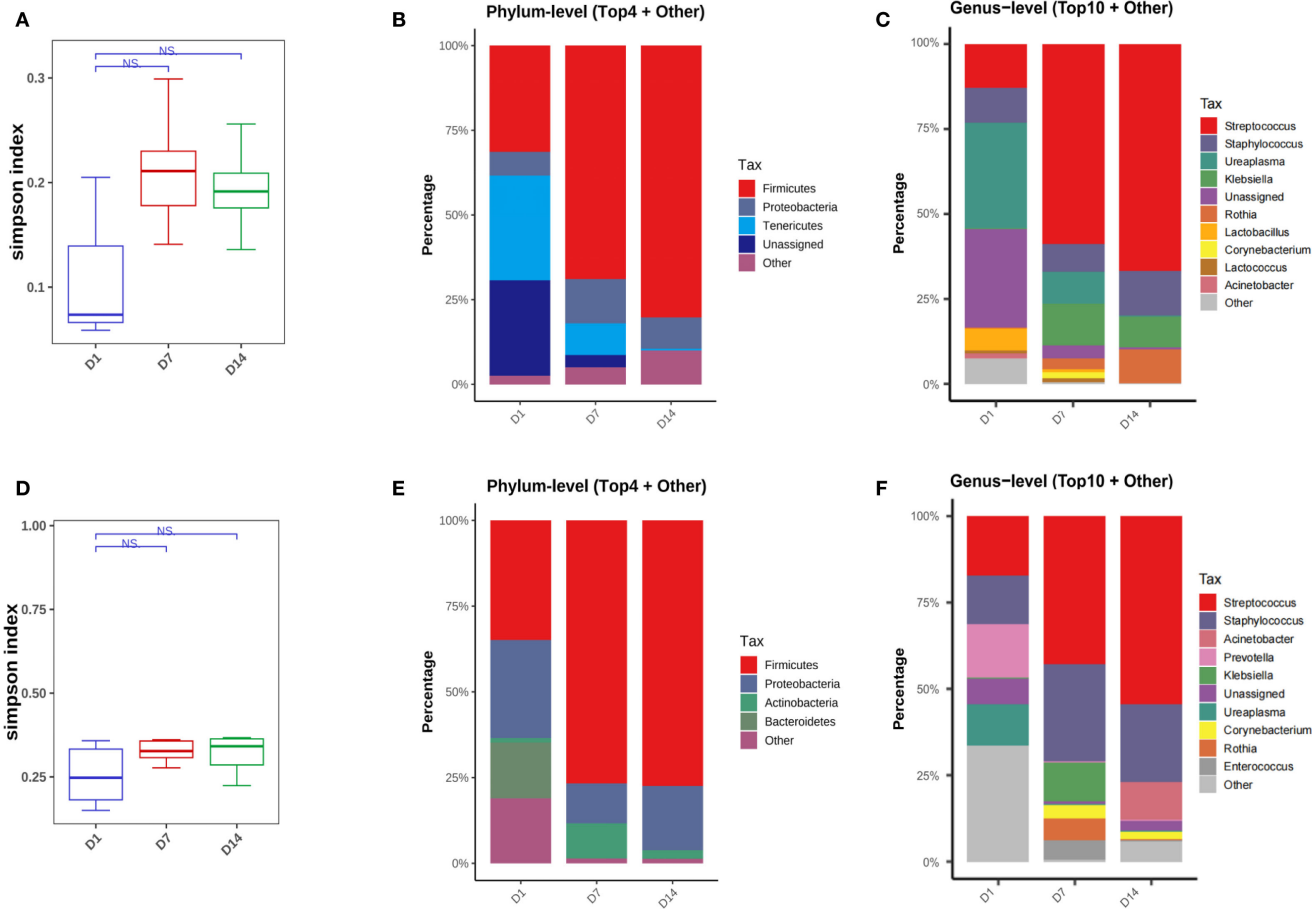
Microbiome dynamics in infants with and without BPD. **(A)** Box plots of simpson index of the non-BPD group at intubation (D1), day 7 post-intubation (D7) and day 14 post-intubation (D14); **(B)** The four species with the highest abundance at the phylum level were identified to create a histogram representing their relative abundance of the non-BPD group at D1, D7 and D14; **(C)** The top ten species with the highest abundance at the genus level were identified to create a histogram representing their relative abundance of the non-BPD group at D1, D7 and D14; **(D)** Box plots of simpson index of the BPD group at intubation; **(E)** The four species with the highest abundance at the phylum level were identified to create a histogram representing their relative abundance of the BPD group at D1, D7 and D14; **(F)** The top ten species with the highest abundance at the genus level were identified to create a histogram representing their relative abundance of the BPD group at D1, D7 and D14. *P < 0.05. **P < 0.01.

There was an increasing trend in bacterial diversity from day 1 to day 7 and the microbial community composition changed over time in the non-BPD group, although no statistically significant differences were found in the Simpson index (**Figure 4A**, **Supplementary Figure 1A**). No significant differences were found in the Simpson index or β-diversity of the BPD infants (**Figure 4D**, **Supplementary Figure 1B**). Analysis at the phylum level revealed that, in both two groups, there was a notable increase in *Firmicutes* in the first two weeks after birth. In the non-BPD group, there was a decrease in *Tenericutes*; while in the BPD group, there was a decrease in *Bacteroidetes* and *Proteobacteria* over time (**Figures 4B, E**). At the genus level, the results indicated that neonates in the non-BPD group were mainly colonized with *Ureaplasma* (*Tenericutes*), *Streptococcus* (*Firmicutes*), and *Staphylococcus* (*Firmicutes*) at birth (**Figure 4C**). Over time, the relative abundance of *Streptococcus* increased, while the proportion of *Ureaplasma* decreased (**Figure 4C**). In contrast, the BPD group were mainly colonized with *Prevotella* (*Bacteroidetes*), *Streptococcu*s (*Firmicutes*), and *Staphylococcus* (*Firmicutes*) at birth. Over time, the proportion of *Streptococcus* and *Acinetobacter* increased, while the abundance of *Prevotella* decreased (**Figure 4F**). MetaCyc pathway analysis based on PICRUSt2 indicated a notable increase in L-lysine biosynthesis and pentose phosphate pathways in the BPD infants.

## Discussion

Growing evidence substantiates the concept of an active interplay between the airway microbiota and pulmonary health (Kayongo et al., 2022; Natalini et al., 2023). The results of this investigation revealed the presence of various bacterial taxa within the respiratory secretions of intubated premature infants, with these microorganisms being identifiable from the moment of birth. The dominance of *Firmicutes* and *Proteobacteria* in the BPD group at intubation, compared with the predominance of *Firmicutes* and *Tenericutes* in the non-BPD group, highlights the potential role of specific microbial taxa in influencing the risk of developing BPD, consistent with the previous studies (Lohmann et al., 2014; Lal et al., 2018). This study adds value through its longitudinal design with specific sampling time points in the preterm infants, capturing dynamic changes of airway microbiota early in life. The key observation was the notable differences over time in the respiratory microbiomes of infants who later developed BPD versus those who did not, as measured by α-diversity and β-diversity. Infants with BPD exhibited decreased Shannon index values and increased Simpson index values, suggesting reduced species richness with a relatively uniform distribution, potentially dominated by a limited number of taxa. Consistent with previous studies, the reduced bacterial diversity observed in the BPD cohort of the study, suggests a potential disruption in microbial homeostasis that may contribute to the pathophysiology of BPD (Lohmann et al., 2014). This is consistent with findings that suggest a varied microbiome plays a crucial role in the development of the immune system and the maintenance of respiratory health in newborns (Pirr et al., 2025).

Moreover, the evolution of the microbiome over the first two weeks of life provides critical insights into the dynamic nature of microbial colonization in preterm infants, suggesting that the microbial landscape is shaped not only by initial colonization but also by subsequent environmental and physiological factors. It has been reported that pulmonary microbial diversity may be affected by invasive mechanical ventilation (Liu et al., 2022). Specifically, high oxygen (O_2_) inhalation-induced oxygen toxicity may reduce the diversity of the pulmonary microbiota, potentially leading to poor prognosis. Moreover, fluctuations in localized O_2_ levels may decrease the variability of the pulmonary microbiome by affecting the immune micro-environment within the lungs (Liu et al., 2022). In mice subjected to hyperoxic conditions, there was a marked alteration in the relative abundance of pulmonary bacteria when compared to those maintained under normoxic conditions, accompanied by a significant reduction in bacterial diversity (Ashley et al., 2020). It can be concluded that elevated oxygen levels during invasive mechanical ventilation may influence the diversity of microbiota by affecting the proliferation rates of intricate microorganisms within the pulmonary microbiota. This finding necessitates additional investigations both *in vitro* and in clinical settings (Ashley et al., 2020). Notably, it is well-documented that antibiotic exposure leads to gut microbiome dysbiosis in early life and prolonged exposure to broad-spectrum antibiotics has been associated with an increased incidence of BPD (Shi et al., 2024). Narrow-spectrum antibiotics including cephalosporin and penicillin were used as empirical antibiotic treatment to prevent infection for the enrolled preterm infants in this study. The results indicated that the infants with BPD received prolonged antibiotic treatment compared to controls, which may alter the lung microbiome and immune responses, potentially increasing the risk of BPD development, but the precise mechanism was not clear. The MetaCyc pathway analysis of the airway microbiome in this study showed an significant elevation in the pentose phosphate pathway in the BPD infants. The over-activation of the pentose phosphate pathway has been reported to promote BPD development (Gong et al., 2021).

In preterm infants of the study, both groups exhibited dominance of *Staphylococcus* and *Ureaplasma* genera in the early airway microbiome, consistent with previous studies (Pammi et al., 2019). Preterm delivery is a primary contributor to neonatal morbidity and mortality worldwide, with *Ureaplasma* species being the most commonly identified microorganisms in amniotic fluid and placental samples in such cases (Pammi et al., 2019). The presence of *Ureaplasma* colonization has been associated with various adverse outcomes, including infertility, stillbirth, histological chorioamnionitis, and several neonatal complications such as congenital pneumonia, BPD, meningitis, and perinatal mortality (Sprong et al., 2020). *Ureaplasma* was abundant at birth but reduced over time in both groups. Prior research has indicated that *Ureaplasma* contributes distinctly to the development of BPD. Nevertheless, recent meta-analyses have failed to deliver uniform evidence to validate this association (Van Mechelen et al., 2023). The existing definitions and classifications of BPD, which mainly focus on the need for respiratory support rather than underlying pathophysiological processes and phenotypic variations, present certain limitations (El-Saie et al., 2023). As a result, the exact mechanisms by which *Ureaplasma* infection induces changes in lung development, as well as the pathways leading to different BPD phenotypes, require further research to enhance our understanding.

The evolutionary trajectory of the microbiome in infants with BPD showed significant divergence from that of their non-BPD counterparts. This study showed that a decreased relative abundance of the phyla *Bacteroidetes* and *Proteobacteria* over time, along with an increased relative abundance of *Firmicutes*, may be associated with BPD progression, consistent with the research by Pablo Lohmann et al (Lohmann et al., 2014). This shift in microbial composition may reflect altered metabolic capacity and could potentially influence the inflammatory processes associated with BPD (Tanabe et al., 2025). In contrast to this study, Charitharth Vivek Lal and colleagues discovered that the airway microbiome in infants with BPD exhibited a higher prevalence of *Proteobacteria*, alongside a reduced presence of *Firmicutes* (Lal et al., 2016). Differences in the clinical characteristics of the patients, variations in the methodologies used, or disparities in the environmental ecology of the research sites may explain this discrepancy. Furthermore, The results indicated an increased proportion of *Streptococcus* (facultative anaerobe) and *Acinetobacter* (aerobe) genera at day 14 of life, which may suggest the presence of ventilator-associated pneumonia or longer oxygen supplementation (Lu et al., 2014). *Streptococcus* might promote BPD development via TLR signaling and MyD88-dependent signaling pathways (Park et al., 2019; Diaz-Dinamarca et al., 2023). Furthermore, a decrease in *Prevotella* was observed in BPD infants. The genera *Prevotella* can produce short-chain fatty acids, which have been demonstrated to be involved in the development and progression of BPD (Li et al., 2020; Gao et al., 2024). These findings raise questions about the potential pathogenic roles these organisms may play in BPD, highlighting the need for further investigation into their contributions to disease progression. In contrast, the dominance of specific genera in infants who did not develop BPD may suggest a protective role in preventing the condition. Additionally, we noted a significant decrease in *Lactobacillaceae* at day 7 post-birth. Recent data has shown that supplementation with *Lactobacillus* strains can improve lung architecture and functionality, diminish the influx of neutrophils, and lower various pro-inflammatory markers of BPD in mice models, likely mediated by the matrix metalloproteinase/proline-glycine-proline pathway (Nicola et al., 2024).

The strengths of this study lie in its thorough approach to characterizing the microbiome of infants with and without BPD during key early developmental stages, thereby providing the potential of leveraging microbiome biomarkers for early BPD risk prediction and developing targeted probiotic interventions. By utilizing a well-designed cohort that includes both the BPD group and the non-BPD group, the study effectively accounts for potential confounding factors such as GA, BW, and sex distribution, thereby improving the reliability of the findings. Advanced microbiome analysis methods, such as Shannon and Simpson diversity indices and LEfSe, offer a detailed understanding of microbial community dynamics and their potential role in BPD development. Additionally, the longitudinal analysis of microbial diversity from birth to day 14 provides valuable insights into how microbial populations evolve over time in relation to BPD. This comprehensive approach not only enhances the robustness of the results but also adds to the growing body of evidence connecting early microbial exposure with respiratory outcomes in preterm infants.

### Limitations

The pilot study had certain limitations. Notably, the PICRUSt2 predictions are inherently speculative, so the connection between TA microbiome dynamics and the underlying mechanisms contributing to the progression of BPD remains to be clarified. The delivery mode is a well-established factor influencing the initial microbial colonization of the neonates (Kennedy et al., 2025). Although subgroup analysis was not feasible in our cohort due to sample size constraints, this potentially confounding variable should be carefully controlled for or stratified in larger, prospective multi-center studies to better elucidate its specific impact on the developing airway microbiome and BPD pathogenesis. The composition of the oral microbiome may partially influence the tracheal microbiome in the intubated premature infants in early life ((Brewer et al., 2021). There was also a lack of data on the intake of human milk and its potential impact on the development of gut and oral microbiome, particularly in relation to the gut-lung axis. In addition, the 16S rRNA sequencing method poses challenges in resolving microbial communities at both the species and strain levels. Future research should explore the potential of shotgun metagenomic approaches to offer a more comprehensive characterization of microbiota composition. Furthermore, the broader community-level interactions, particularly how the airway microbiome produces metabolites that influence host immunity in BPD, remain under-explored and require further investigation.

## Conclusions

Collectively, these findings highlight the crucial role of the microbiome in the pathophysiology of BPD and suggest that further exploration of microbial interventions could offer the potential as a novel targeted therapeutic strategy in the BPD treatment. Future research should focus on investigating the relationship between the airway metabolome and microbiome in the context of respiratory diseases.

## Data Availability

The data presented in the study are deposited in the NCBI SRA repository. Accession to cite for these SRA data: PRJNA1337180. https://www.ncbi.nlm.nih.gov/sra/PRJNA1337180.
