# Supplementary material for: Characterization of the airway microbiome in preterm infants with bronchopulmonary dysplasia
Source: Front Cell Infect Microbiol. 2025 Oct 13;15:1654502. doi: 10.3389/fcimb.2025.1654502 (PMC12554745; doi:10.3389/fcimb.2025.1654502)
Supplement: Supplementary file 1 [file Table1.doc]

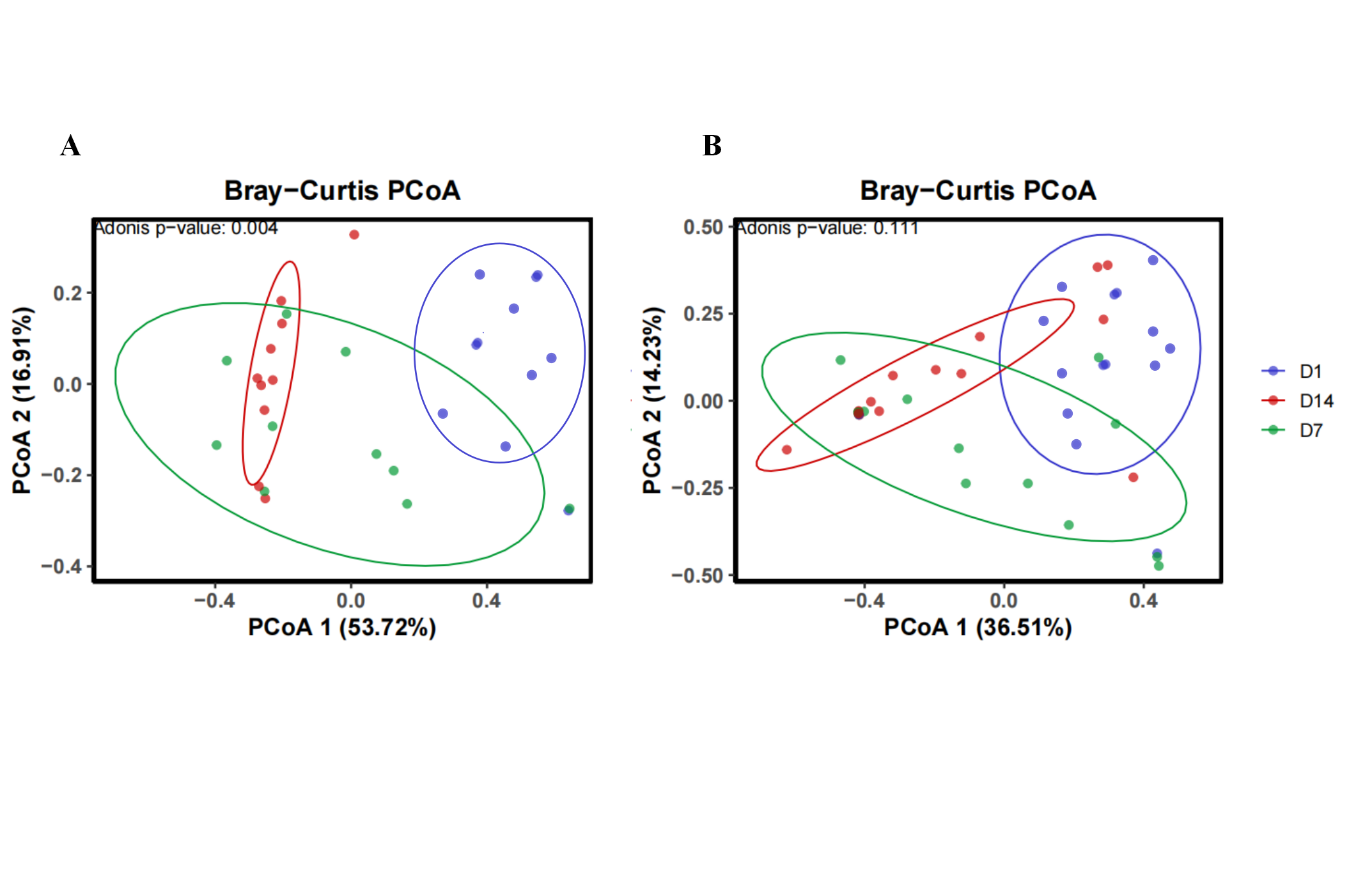


**Supplementary Figure 1 Microbiome dynamics in infants with and without BPD**

Principal coordinate analysis (PCoA) of the microbiota based on the Bray-Curtis distance between the non-BPD group (A) and the BPD group (B) .
